# Immune Responses to the Cancer Testis Antigen XAGE-1b in Non Small Cell Lung Cancer Caucasian Patients

**DOI:** 10.1371/journal.pone.0150623

**Published:** 2016-03-03

**Authors:** Kanako Saito, Eiichi Nakayama, Danila Valmori

**Affiliations:** 1 Institut National de la Santé et de la Recherche Médicale, Unité 1102, Equipe Labellisée Ligue Contre le Cancer, Institut de Cancérologie de l’Ouest, Nantes-Saint Herblain, France; 2 Faculty of Health and Welfare, Kawasaki University of Medical Welfare, Kurashiki, Japan; 3 Faculty of Medicine, University of Nantes, Nantes, France; Mie University Graduate School of Medicine, JAPAN

## Abstract

Immunotherapy approaches using checkpoint blockade, alone, or in combination with tumor antigen vaccination, or adoptive cell transfer, are emerging as promising approaches for the treatment of non-small cell lung cancer (NSCLC). In preparation for upcoming combined immunotherapy approaches in NSCLC, here, we have assessed spontaneous immune responses to XAGE-1b, a tumor specific antigen of the Cancer Testis Antigen group that has been previously reported to be spontaneously immunogenic in the Japanese population, in a cohort of Caucasian patients with NSCLC. We found spontaneous serological responses to XAGE-1b in 9% of the patients. Importantly, these responses were limited to, and represented 13% of, patients with adenocarcinoma tumors, the most frequent histological subtype, for which immunotherapy approaches are under development. Using a set of overlapping peptides spanning the entire XAGE-1b protein, and in support of the serological data, we detected significant XAGE-1b specific CD4^+^ T cell responses in all XAGE-1b seropositive patients and identified several CD4^+^ T cell epitopes. Altogether, our results support the relevance of the XAGE-1b antigen in Caucasians NSCLC patients with adenocarcinoma, and the implementation of future immunotherapies exploiting the high immunogenicity of the antigen in this patient population.

## Introduction

Lung cancer is the leading cause of cancer-related mortality worldwide, with non-small cell lung cancer (NSCLC) accounting for approximately 85% of all lung cancer cases [1]. Despite recent improvements in therapeutic strategies, NSCLC constitutes therefore one of the major public health problems. In the majority of cases, symptoms usually appear at an advanced phase of the disease, in the metastatic or locally advanced stages, thus making the treatment difficult [2]. After initial diagnosis, accurate staging is crucial for determining an appropriate therapy. Surgical resection of the tumor is still the standard of care, but, unfortunately, it is applicable and can be considered a consistent and successful option for cure, only in patients with resectable tumors and able to tolerate the resection. However, approximately 70% of lung cancer patients present with locally advanced or metastatic disease at the time of diagnosis [2]. For these patients, the first line of treatment is platinum-based chemotherapy, which has proved to be beneficial for palliation and represents the standard of care. Radiotherapy is also frequently used as a first line of treatment for NSCLC and the administration of concurrent chemotherapy and radiation is indicated for stage III lung cancer [2]. However, even with these treatments, the overall survival rates in NSCLC patients are still dramatically low, with an average 5-year survival rate of 17% in patients with early disease and 4% in patients with metastatic disease [3]. Therefore, there is an urgent need to develop new therapeutic strategies to induce more effective clinical responses and prolong the overall survival in this patients population. In the last decade, new knowledge in cancer biology has opened novel potential therapeutic approaches, including targeted therapies and immunotherapies. Targeted therapies, such as those using angiogenesis inhibitors, epidermal growth factor receptor inhibitors (EGFRi) or tyrosine kinase inhibitors (TKi) can be combined to the main treatment modalities in patients presenting specific mutations [4–6]. However, the proportion of patients expressing these mutations is relatively small (for instance, only 10–15% of NSCLC harbour EGFR mutations). In addition, the clinical effects of these treatments are frequently not long lasting, due to the development of resistance [7,8].

On the other hand, immunotherapeutic strategies have the potential to strengthen the patient’s immune response, to induce stable clinical responses and extend survival [1]. Emerging immunotherapeutic strategies are those using checkpoint blockade specific antibodies, that have shown clinical efficacy in subgroup of patients. Recent data suggest that these responder patients are those that harbour spontaneous immune responses to the autologous tumor. Other immune based strategies in NSCLC include cancer vaccination approaches using Cancer/Testis antigens (CTA) [1,9], proteins encoded by genes normally expressed in germ cells in testis and fetal ovary and, in some cases, in placental trophoblasts, silenced in normal adult tissues, but aberrantly re-expressed in various types of cancer [10,11]. CTA are largely expressed in cancers of different histological subtypes, are often highly immunogenic and are therefore considered among the most attractive targets for the development of cancer vaccines [11]. Cancer vaccines as monotherapy are currently under evaluation in NSCLC and could be effective in patients with minimal residual disease [9]. Despite the first clinical trials applying this type of strategy have not met their clinical endpoint [12], combination of vaccination with checkpoint blockade therapies are very promising [1]. However, the use of these strategies requires the identification of tumor specific antigens expressed by a significant fraction of NSCLC, as well as of the characteristics of the tumors that express them. Various CTA have been shown to be expressed in NSCLC. XAGE-1b is encoded by the XAGE-1 gene, located in the Xp11.22 region of the X chromosome [13,14]. Four transcript variants, XAGE-1a to d, have been identified [14–16]. One transcript expressed in tumors, XAGE-1b, encodes an 81 amino acids long protein [14,17]. XAGE-1b was reported to be the prevalent transcript expressed in NSCLC. XAGE-1b expression was observed in 45% of adenocarcinomas of Japanese patients [18]. XAGE-1b was shown to induce antibody and T cell responses in Japanese NSCLC patients. The frequency of antibody response was reported to be higher in adenocarcinoma patients (14%) than in SCC patients (2%) [19]. The purpose of this study was to extend these findings to the Caucasian population, to evaluate the potential of XAGE-1b-based immunotherapy in Caucasians.

## Materials and Methods

### Patients samples and cell purification

Peripheral blood mononuclear cells (PBMC) samples were collected from a cohort of 141 NSCLC patients (Institut de Cancérologie de l’Ouest (ICO), Saint-Herblain, France), after approval by the Institutional Review Board of the ICO. PBMC from 60 healthy donors were obtained from the Etablissement Français du Sang (EFS) Pays de la Loire, after approval by the Institutional Review Board of the Etablissement Français du Sang Pays de la Loire (Nantes, France). A signed informed consent form was obtained from all donors participating in the study. The cohort included 91 adenocarcinomas, 40 squamous cell carcinomas (SCC) and 10 NSCLC tumors of other subtypes. PBMC were isolated by density gradient centrifugation. CD4^+^ and CD8^+^ T cells were enriched from PBMC by magnetic cell sorting using MicroBeads (MACS Cell separation Technology, Miltenyi Biotec).

### Proteins and peptides

The series of 17 16-mer XAGE-1b overlapping peptides (OLP, Table 1) were synthesized on a Multiple Peptide Synthesizer, AMS422, ABINED at Okayama University. The recombinant NY-ESO-1 protein was produced in *Escherichia coli* as described previously [20]. The XAGE-1b (GAGED2a) protein (81 amino acid long) was synthesized using a peptide synthesizer by GL Biochemistry.

**Table 1:** XAGE-1b peptides.

Peptide	Amino acid position	Sequence
C1	1–16	MESPKKKNQQLKVGIL
C2	5–20	KKKNQQLKVGILHLGS
C3	9–24	QQLKVGILHLGSRQKK
C4	13–28	VGILHLGSRQKKIRIQ
C5	17–32	HLGSRQKKIRIQLRSQ
C6	21–36	RQKKIRIQLRSQCATW
C7	25–40	IRIQLRSQCATWKVIC
C8	29–44	LRSQCATWKVICKSCI
C9	33–48	CATWKVICKSCISQTP
C10	37–52	KVICKSCISQTPGINL
C11	41–56	KSCISQTPGINLDLGS
C12	45–60	SQTPGINLDLGSGVKV
C13	49–64	GINLDLGSGVKVKIIP
C14	53–68	DLGSGVKVKIIPKEEH
C15	57–72	GVKVKIIPKEEHCKMP
C16	61–76	KIIPKEEHCKMPEAGE
C17	65–81	KEEHCKMPEAGEEQPQV

### In vitro stimulation of CD4^+^ and CD8^+^ T cells

Purified CD4^+^ and CD8^+^ T cells were stimulated with irradiated autologous antigen-presenting cells (APCs) in the presence of a mixture of 17 16-mer overlapping peptides (OLP, 2 μM) (Table 1) spanning the entire amino acid sequence of the XAGE-1b protein, rhIL-2 (50 IU/ml) and rhIL-7 (10 ng/ml). Cells were cultured in 96-well culture plates at 37°C (CO_2_ 5%) in Iscove’s Modified Dulbecco’s Medium (IMDM, Gibco) supplemented with 8% heat inactivated human serum (HS), GlutaMAX (Gibco), HEPES (Gibco), non-essential amino acids (MEM NEAA, Gibco), Ciprofloxacine and Penicillin/Streptomycin.

### Intracellular cytokine staining (ICS)

Following *in vitro* stimulation, CD4^+^ and CD8^+^ T cells were cultured for 14 days and then assessed for XAGE-1b specific T-cell responses. Cultures were collected and stimulated in the presence or in the absence of the XAGE-1b peptide pool (2 μM) and tested in a standard 4hr intracellular cytokine staining (ICS), using fluorescent-conjugated cytokine-specific antibodies αCD4 (BD Biosciences), αCD8 (BD Biosciences), αIL-17A (eBioscience) and αIFN-γ (BD Biosciences). The cytokine expression profile was then assessed by flow cytometry (FACSAria II, BD Biosciences).

### IFN-γ capture assay and establishment of CD4^+^ and CD8^+^ T cell clones

CD4^+^ and CD8^+^ T cell lines were collected and stimulated in the presence or absence of XAGE-1b peptide pool (2 μM). Stimulated samples were incubated with a bi-specific CD45 and IFN-γ antibody (IFN-γ catch reagent, Miltenyi Biotec) and incubated for 5 minutes on ice. Cells were then placed on a slow rotating device (Miltenyi Biotec) for 45 minutes at 37°C, to allow cytokine secretion. After this period, cells were washed in cold buffer and incubated with the APC-conjugated IFN-γ detection reagent (Miltenyi Biotec), for 15 minutes on ice, in the presence of αCD4 (FITC-conjugated) cytokine-specific antibody. Cells were further washed with cold buffer and analysed by FACS. CD4^+^ T cells that secreted IFN-γ specifically in response to the XAGE-1b peptide pool were sorted by flow cytometry cell, using a FACSAria II. The isolated CD4^+^ IFN-γ producing T cells were cloned under limiting dilution conditions, in 96-well plates, by stimulation with phytohemagglutinin (PHA-L, 1 μg/ml) in the presence of irradiated allogenic PBMC and EBV cell lines (EBV-HV, EBV-LAZ) and rhIL-2 (150 IU/ml). CD4^+^ T cell clones were maintained in IMDM supplemented with 8% HS by re-stimulation every 2–3 weeks.

### Assessment of serological responses

Serological responses were assessed by ELISA, using 96-well plates coated with the XAGE-1b protein (2 μg/ml) or the NY-ESO-1 protein (1 μg/ml) overnight at 4°C. Sera were assessed in a range of dilutions from 1/100 to 1/100,000. Antibody titers were calculated as the serum dilution corresponding to 50% of the maximal optical density response.

### Assessment of IFN-γ production by ELISA

To assess antigen specific IFN-γ production, XAGE-1b specific clones were stimulated in the presence or in the absence of the peptide pool (50 × 10^3^ per well, in 96-well round-bottom culture plates). IFN-γ was measured by ELISA in 24 h culture supernatants, using Nunc Maxisorp flat-bottom 96-well plates. For the fine specificity assay, cells (50 × 10^3^ per well) were stimulated in the presence or in the absence of XAGE-1b single peptides (300 μM), and IFN-γ concentration was measured as above. For the antibody blocking experiments, αDP (1 μg/ml), αDQ (1 μg/ml), αDR (1 μg/ml) and αDR52b (ascites, 1:10 dilution) antibodies were added to the assay culture. The HLA-DR restricting alleles were similarly assessed by IFN-γ ELISA, using molecularly defined APC. LDR1, kindly provided by Dr. Hassan M. Zarour (University of Pittsburgh, USA), were maintained in complete RPMI medium and checked for HLA-DR expression. EBV149 and EBV156, (Epstein-Barr virus transformed lymphoblastoid cell lines) were derived from patients expressing HLA-DRB1*13.

## Results

### Serological responses to XAGE-1b and NY-ESO-1 in NSCLC patients

**Fig 1:**
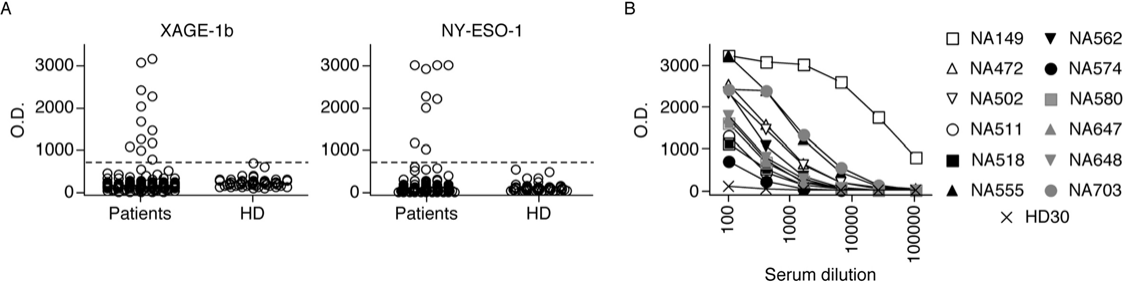
Assessment of serological responses to XAGE-1b and NY-ESO-1 NSCLC patients. (A) Summary of the ELISA for the 141 NSCLC patients and 60 healthy donors (HD). The Optical density (OD) cut-off between responders and non-responders was determined as the mean OD obtained with the sera from the 60 HD + 5 standard deviations (SD) (dotted line). (B) Sera titration from the 12 XAGE-1b seropositive patients and from 1 HD was performed in a range of dilutions, from 1/100 to 1/100,000. Antibody titers were determined as the serum dilution corresponding to 50% of the maximal OD response.

We screened a cohort of 141 NSCLC patients for serological responses to XAGE-1b and NY-ESO-1, as internal control, by ELISA. The results of the screening are summarized in Fig 1. We first assessed the sera from patients along with those of 60 healthy donors at a dilution of 1:100. We detected significant serological responses to XAGE-1b in 12 patients (Fig 1A). In contrast, antibody responses to NY-ESO-1 were detected in 9 patients. We then assessed the sera from the 12 XAGE-1b seropositive patients in a titration curve and calculated the antibody titer, defined as the serum dilution giving 50% of the maximal response. The titers obtained were variable among the seropositive patients and ranged from 1:250 to 1:40000 (Fig 1B).

In the cohort, 91 tumors were adenocarcinomas, 40 were squamous cell carcinomas (SCC) and 10 belonged to other subtypes. XAGE-1b seropositive patients were exclusively found in the adenocarcinoma subgroup, and represented 13% of the subgroup (Table 2). In contrast, NY-ESO-1 seropositive patients belonged mostly to the SCC subgroup, and represented 13% of the subgroup whereas only 3% of the adenocarcinoma patients had significant serological responses to NY-ESO-1 (Table 2). Thus, we confirmed that XAGE-1b is spontaneously immunogenic in NSCLC, mostly in adenocarcinomas, and demonstrated that this is the case not only in the Japanese population as described previously, but also in Caucasians. The correlation between serological responses to XAGE-1b and adenocarcinoma histological subtype reached statistical significance (Fisher’s exact test, P = 0.0176). In contrast, our findings indicate that antibody responses to NY-ESO-1 are more frequent in SCC patients, with data close to, but not yet reaching statistical significance (Fisher’s exact test, P = 0.0563). These results are in line with previously published data that seropositivity of NY-ESO-1 was observed in 29% and 15% of SCC and adenocarcinoma patients, respectively [21].

**Table 2:** Serological responses to XAGE-1b and NY-ESO-1 in relation to NSCLC histological types.

	Total	XAGE-1b Ab^+^ ^c^	*P* ^d^	NY-ESO-1 Ab^+^ ^c^	*P* ^d^
NSCLC ^a^	141	12 (9%)		9 (6%)	
Adenocarcinoma	91	12 (13%)	*0*.*0176*	3 (3%)	*0*.*0563*
SCC ^b^	40	0		5 (13%)	
Other	10	0		1 (10%)	
					

^a^ Non-small-cell lung cancer.

^b^ Squamous cell carcinoma.

^c^ Serological responses to the antigens were assessed in patients sera as shown in Fig 1.

^d^ Statistical significance of the correlation between serological responses to XAGE-1b and NY-ESO-1 and tumors’ histological subtype was determined using Fisher’s exact test. Statistical analyses were performed taking into account the adenocarcinoma and SCC groups only.

### Assessment of XAGE-1b-specific CD4^+^ and CD8^+^ T cell responses

**Fig 2:**
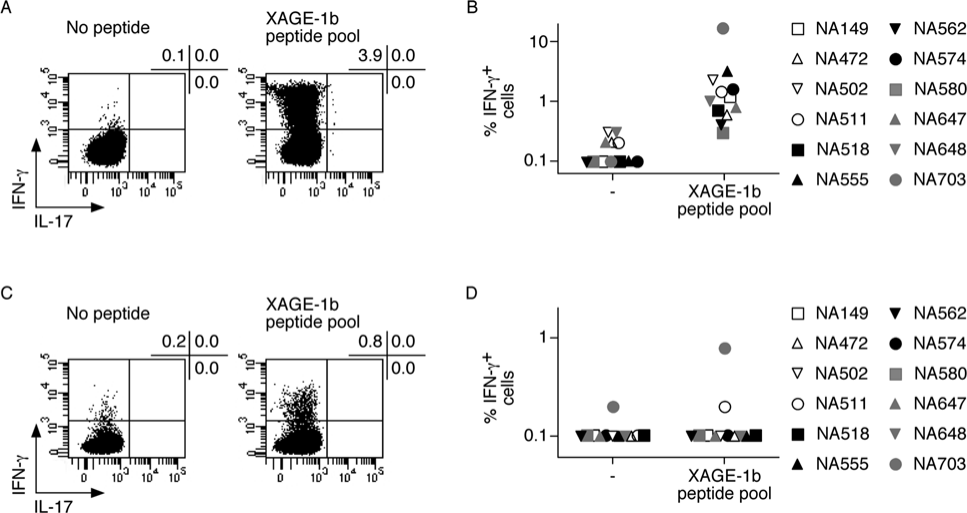
Assessment of circulating CD4^+^ and CD8^+^ T cell responses to XAGE-1b in NSCLC patients. (A, C) The presence of XAGE-1b specific CD4^+^ (A) and CD8^+^ (C) T cell responses was assessed in peptide pool stimulated cultures by intracellular cytokine staining, with cytokine specific antibodies αIFN-γ and αIL-17, after stimulation in the absence or the presence of the XAGE-1b peptide pool. The percentages of IFN-γ producing cells are shown in the upper left quadrants of each dot plot, showing increased IFN-γ production in peptide stimulated samples. The data shown are an example of a CD4^+^ and CD8^+^ T cell responder patient. (B, D) Summary of the CD4^+^ and CD8^+^ T cell responses are shown for all the 12 seropositive patients. As shown in (B), CD4^+^ responses are detected for all seropositive patients, whereas a significant CD8^+^ response was identified only in 1 patient, NA703 (D). Values at least 3-fold higher than baseline (no peptide) were considered significant.

Based on the results of the serological screening, we assessed the 12 antibody responder patients for CD4^+^ and CD8^+^ T cell responses to XAGE-1b. To this end, we enriched CD4^+^ and CD8^+^ T cells from PBMC of the patients by magnetic cell sorting and stimulated them in the presence of a pool of 17 16-mer overlapping peptides, spanning the complete 81 amino acid sequence of the XAGE-1b protein (Table 1) and cultured them for 14 days in the presence of irradiated autologous APC, rhIL-2 and rhIL-7. At the end of the culture period, we stimulated aliquots of the cultures derived from each patient in the absence or in the presence of the XAGE-1b peptide pool and assessed responsiveness to XAGE-1b in a 4hr standard intracellular cytokine staining assay, using antibodies specific for IFN-γ and IL-17. We analysed the samples by flow cytometry (Fig 2A and 2C) and compared the proportion of IFN-γ producing cells obtained for each culture in relation to it’s own baseline response (in the absence of peptides) to confirm the antigen-specific response. CD4^+^ T cell responses were considered significant when the proportion of CD4^+^ T cells producing IFN-γ in the presence of the peptide pool was at least 3-fold higher than that observed at baseline. Based on these criteria, we detected significant CD4^+^ T cell responses to XAGE-1b in all seropositive patients (Fig 2B), In contrast, only in the case of 1 patient, we detected a XAGE-1b specific CD8^+^ T cell response (Fig 2D). However, we failed to detect specific production of IL-17 in response to XAGE-1b for any of the cultures.

### Generation of CD4^+^ T cell clones, assessment of the active peptides and HLA restriction

**Fig 3:**
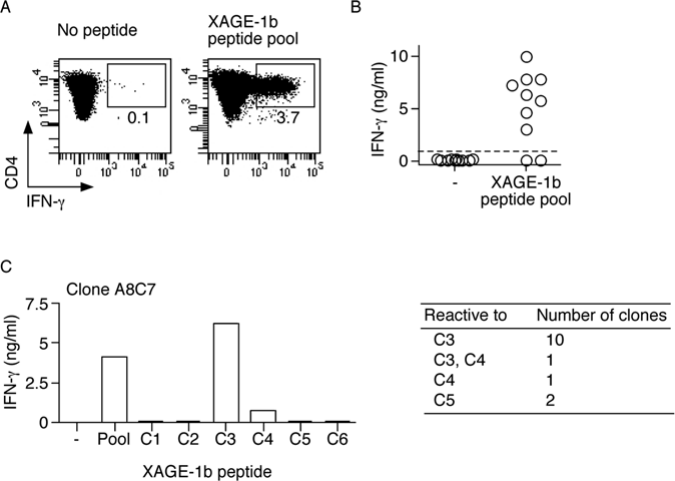
Isolation of XAGE-1b specific CD4^+^ T cells and generation of specific clonal populations. (A) XAGE-1b specific CD4^+^ T cells were isolated by IFN-γ capture assay. Numbers indicated in the upper right region of dot plots show the percentages of IFN-γ producing cells among the CD4^+^ T cells, after stimulation in the absence or in the presence of the peptide pool. IFN-γ producing cells were sorted and cloned under limiting dilution conditions. (B) CD4^+^ T-cell clones were obtained and screened to assess reactivity to XAGE-1b. Clones were stimulated in the presence or the absence of the peptide pool and specificity to XAGE-1b was determined by ELISA. Responses were considered significant when the amount of IFN-γ detected in the presence of peptide was at least 3-fold higher than that detected in the absence of peptide. (C) Aliquots of XAGE-1b specific CD4^+^ T-cell clones were stimulated in the absence or presence of the peptide pool or of individual single peptides and the amount of IFN-γ was measured in 24-hour culture supernatants by ELISA. (D) Reactivity to XAGE-1b single peptides was assessed in 14 clones and 4 fine specificities were identified.

**Fig 4:**
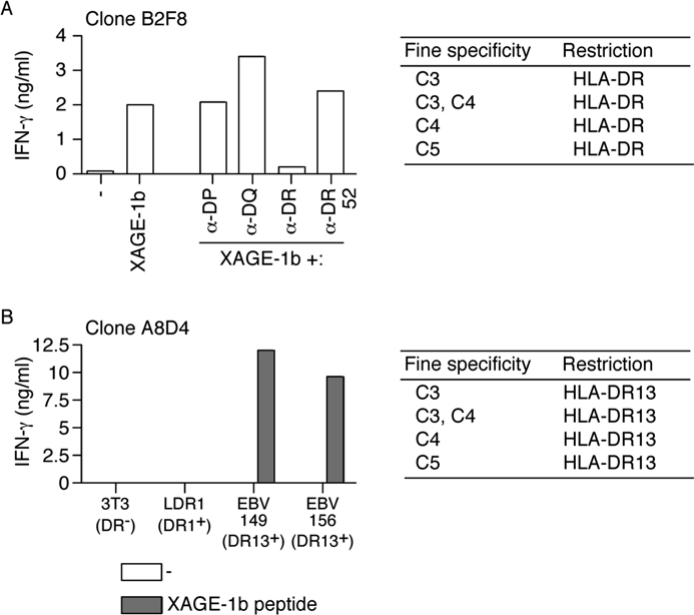
Assessment of the HLA restriction of XAGE-1b-specific CD4^+^ T-cell clones. (A) Identification of MHC II restricting molecules. The left panel shows an example of an HLA-II restriction assay clone, B2F8. Peptide recognition was assessed by ELISA, incubating XAGE-1b specific CD4^+^ T-cell clones exhibiting each of the 4 fine specificities either in the absence or in the presence of αDP, αDQ, αDR and αDR52b specific antibodies. Peptide recognition was restricted by HLA-DR in all cases, as summarized in B. The HLA-DR restricting allele was identified by incubating XAGE-1b specific CD4^+^ T-cell clones with different APC including untransfected (3T3) or DR1-transfected mouse fibroblast (LDR1) and DR13-expressing EBV-cell lines (EBV149, EBV 156). Each APC was pre-incubated with the reactive peptides and the restricting allele was determined by assessing their capacity to present the peptides to the corresponding CD4^+^ T-cell clones. Peptide recognition was assessed by measurement of IFN-γ secretion in culture supernatants, in the presence of each APC. As summarized, we found that peptide recognition was restricted by HLA-DR13 in all cases.

We isolated XAGE-1b reactive CD4^+^ T cells from one of the XAGE-1b seropositive patients, NA555, by enriching the fraction of cells that secreted IFN-γ specifically in response to the XAGE-1b peptide pool, by Miltenyi IFN-γ capture assay (Fig 3A). To obtain specific clones, we cultured the isolated cells under limiting dilution conditions, expanded them and then assessed them in the absence or in the presence of the XAGE-1b peptide pool (Fig 3B). We obtained 14 XAGE-1b specific CD4^+^ T cell clones, that we characterized for their fine specificity by assessing reactivity towards the 17 overlapping single peptides composing the XAGE-1b peptide pool. We initially tested the single peptides in subpools of 3 peptides each and determined that the reactive peptides were localized in the first 2 subpools including peptides C1-C3 and C4-C6 (Table 1). We then assessed the response to the single peptides included in those regions. Fig 3C (left panel) shows an example of this analysis for a XAGE-1b reactive CD4^+^ T-cell clone (clone A8C7) that recognized peptide C3. A summary of the fine specificity assessment of the 14 CD4^+^ T-cell clones is shown in Fig 3C (right panel). 10/14 clones were reactive to peptide C3, 1 clone recognized peptides C3 and C4, 1 clone recognized peptide C4, and 2 clones recognized peptide C5. Altogether, we found 4 different fine specificities. Based on this reactivity, we determined the MHC class II molecules restricting antigen recognition of single clones representative of the 4 fine specificities. To this end, we assessed antigen recognition in the presence of αDP, αDQ, αDR and αDR52b specific antibodies, in the context of an IFN-γ secretion assay, where each clone was tested in the presence or in the absence of the reactive peptide. Fig 4A shows an example of an MHC class II restriction assay performed with the CD4 T-cell clone B2F8, which recognized peptide C3. As shown, we determined that antigen recognition by the clone was specifically inhibited by the αDR antibody, while no inhibition was detected with αDP and αDQ blocking antibodies, indicating that this clone was restricted by HLA-DR. Similar results were obtained for the other clones. We then aimed at identifying the HLA-DR restricting allele. HLA-DR molecular typing revealed that NA555 expressed the DRB1*01 and DRB1*13 alleles. To determine which of these molecules was the restricting allele, we stimulated the CD4^+^ T-cell clones in the presence of LDR1 cells (mouse fibroblasts transfected with HLA-DR1) or EBV-transformed B-cell lines (EBV 149, EBV 156) expressing the DR13 allele. We pre-incubated APC with the reactive peptides and assessed their capacity to present the corresponding XAGE-1b peptide to the CD4 T-cell clones. Responses were determined by measuring the amount of IFN-γ in 24-hour culture supernatants, by ELISA. For all clones, we detected peptide recognition in the presence of the EBV-B cell lines (EBV 149, EBV 156) expressing DRB1*13 but not in the presence of LDR1 cells (Fig 4B).

## Discussion

In this study, we investigated spontaneous antibody and T cell responses to the CTA XAGE-1b in a cohort of 141 Caucasian NSCLC patients. Our findings demonstrate that serological responses to XAGE-1b are largely restricted to adenocarcinomas as they are found in about 13% of them but are not detected in SCC. Taking into account that XAGE-1b has been reported to be expressed in about 45% of adenocarcinomas [18] (but only 7% of SCC), serological responses seem rather frequent in this group of patients, being found in about one third of them. These results are in line with previously published data in the Japanese population, reporting serological responses to XAGE-1b in 14% of adenocarcinomas but in only 2% of SCC patients [19]. Our data also indicate that serological responses to NY-ESO-1 are instead more frequent in SCC than in adenocarcinoma, as they are found in 13% of SCC but only in 3% of adenocarcinomas from Caucasians. Again this is in line with results from previous studies that have reported more frequent spontaneous serological responses to NY-ESO-1 in SCC than in adenocarcinoma [21]. Similar to NY-ESO-1, we have recently reported that the MAGE-A antigens, that also belong to the CTA group, were more frequently expressed in SCC, as compared to adenocarcinoma [22]. Together, our findings confirm XAGE-1b immunogenicity in lung adenocarcinoma, highlighting the importance of this CTA as a promising target for immunotherapy against this tumor subtype.

In our study, we detected significant XAGE-1b-specific CD4^+^ T cell responses in all seropositive patients, again in line with previous data in the Japanese population [19]. We established clonal populations from a high responder, determined that the reactivity of the clones was towards sequences located in peptides C3-C5, that correspond to the 9–32 amino acid region of the protein, and identified 4 fine specificities of antigen recognition. We demonstrated that, for all fine specificities, peptide recognition was restricted by HLA-DR. By assessing antigen presentation using APC that share single HLA-DR alleles with those of the patient, we determined that antigen recognition by the clones was restricted by the DRB1*13 allele. It is noteworthy that, whereas other XAGE-1b MHC class II epitopes restricted by other alleles have been reported [19,23,24], DRB1*13 restricted epitope described here has not been previously reported.

Some of our results are at variance with those reported in literature [19,25]. On one hand, both in our study and in previous studies in the literature [19] XAGE-1b specific CD4^+^ T cells responses were detected in virtually all NSCLC XAGE-1b seropositive patients. However, at variance with the results of a recently reported study [25] in our study, XAGE-1b specific CD4^+^ T cells produced IFN-γ but not IL-17, and thus exhibited a clear Th1 profile. In addition, we detected a significant CD8^+^ T cell response in only one of the 12 seropositive patients, which is much less frequent than the frequency of 6/9 seropositive patients previously reported in the Japanese cohort [19]. This discrepancy could be at least partially explained by the different methodologies used that may significantly differ in terms of sensitivity and specificity of detection. Indeed, whereas we assessed CD8^+^ T cell responses in a 4hr standard intracellular cytokine staining (ICS) assay, in the Japanese study, CD8^+^ T cell responses to XAGE-1b were assessed by IFN-γ secretion capture assay [19]. Thus, either the frequency of XAGE-1b specific CD8^+^ T cell responses in seropositive patients has been underestimated in our study, because of the lower sensitivity of the method used, or overestimated in the study from Ohue Y et al. because of the lower specificity of the IFN-γ capture assay, two hypotheses that are not mutually exclusive. However, in favour of an over-estimation of the number of true XAGE-1b CD8^+^ T cell responses in the Japanese study, is the fact that the IFN-γ secretion levels obtained in that study in most of the CD8^+^ T cell cultures upon stimulation with XAGE-1b peptides was very low, close to background levels [19].

In conclusion, altogether, our results support the relevance of the XAGE-1b antigen in Caucasian patients with lung adenocarcinoma and encourage the implementation of future immunotherapies exploiting the high immunogenic of this antigen in this patients’ population.

In addition, it is noteworthy that, because the expression XAGE1b is known to be regulated by DNA methylation, DNA methyltransferase inhibitors could increase the potential patient population eligible for XAGE-1b directed immunotherapies, as previously reported in the case of NY-ESO-1 [26]. Exploring this possibility could be the subject of future studies.
