# Correction: Immune Responses to the Cancer Testis Antigen XAGE-1b in Non Small Cell Lung Cancer Caucasian Patients

**DOI:** 10.1371/journal.pone.0160538

**Published:** 2016-07-28

**Authors:** Alessandro Sardaro, Kanako Saito, Eiichi Nakayama, Danila Valmori

Dr. Alessandro Sardaro should be included in the author byline. He should be listed as a co-first author with Kanako Saito, and his affiliation is: Institut National de la Santé et de la Recherche Médicale, Unité 1102, Equipe Labellisée Ligue Contre le Cancer, Institut de Cancérologie de l’Ouest, Nantes-Saint Herblain, France. The contributions of this author are as follows: Performed the experiments, analyzed the data, wrote the paper.

The correct citation is: Sardaro A, Saito K, Nakayama E, Valmori D (2016) Immune Responses to the Cancer Testis Antigen XAGE-1b in Non Small Cell Lung Cancer Caucasian Patients. PLoS ONE 11(3): e0150623. doi:10.1371/journal.pone.0150623
